# Comparing the effectiveness of twine- and binder-seeding in the Laminariales species *Alaria esculenta* and *Saccharina latissima*

**DOI:** 10.1007/s10811-020-02069-5

**Published:** 2020-03-03

**Authors:** Philip D. Kerrison, Mairi Innes, Adrian Macleod, Emily McCormick, Peter D. Elbourne, Michele S. Stanley, Adam D. Hughes, Maeve S. Kelly

**Affiliations:** 1 Scottish Association for Marine Science (SAMS), Scottish Marine Institute, Dunbeg, Argyll, 1QA UK; 2 New Wave Foods Ltd, 1 Averon Way, Alness, IV17 0PF UK

**Keywords:** *Saccharina latissima*, *Alaria esculenta*, Phaeophyceae, Binder, Seeding, Morphology, Density

## Abstract

The continuing expansion of seaweed cultivation could assist in ensuring future global food security. The Laminariales species *Alaria esculenta* and *Saccharina latissima* are each cultivated for food across their European ranges. The predominant method for cultivating European kelps involves growing juveniles on twine within a hatchery which is then deployed at a farm site. The associated hatchery and deployment cost of this approach are relatively high. A new and innovative methodology—called binder-seeding—can reduce these costs, but, has yet to be validated. We compare the biomass yield and morphology of *A. esculenta* and *S. latissima* cultured using either the traditional twine-longline method or binder-seeding onto AlgaeRope and AlgaeRibbon, specially designed textiles. In a controlled growth experiment, *A. esculenta* had a similar biomass yield on all materials, but fronds were shorter (23 ± 7%) and thinner on the AlgaeRibbon (42 ± 4%) due to a 3–4-fold higher density of developing sporophytes compared to the twine-longline. In contrast, *S. latissima* gave a 4-fold higher biomass yield on the AlgaeRibbon in June (4.0 kg m^−1^), but frond morphology was not different between materials, despite a 4-fold higher sporophyte density on the AlgaeRibbon. The stipe length of both species also increased at the higher sporophyte density on the AlgaeRibbon. The AlgaeRope gave an intermediate response or was similar to the twine-longline. These results show that binder-seeding onto the AlgaeRibbon significantly increases the achieved biomass yield in *S. latissima.* These results can assist cultivators to select the most appropriate method of kelp cultivation depending on morphological/yield requirements of the end use. Further study is needed on the optimisation of the binder-seeding density and its impact on thallus morphology.

## Introduction

Increasing the production of low trophic food is essential to ensure future food security as the global population continues to rise (FAO 2017). Macroalgae represent only 0.3% of total food production, but there is a room for expansion of production in the marine environment, whereas terrestrial agriculture is significantly constrained by space (Forster and Radulovich 2015). In the North Atlantic, macroalgae are a relatively underexploited resource for a range of end uses including human food, animal feed, chemical extracts, cosmetics and bioactives (Holdt and Kraan 2011; Kraan 2013; Bleakley and Hayes 2017). There is a growing macroalgae aquaculture industry across Europe (Freitas et al. 2016; Peteiro et al. 2016; Stévant et al. 2017). The Northern Atlantic kelp species *Saccharina latissima* (Linnaeus) C.E. Lane, C. Mayes, Druehl & G.W. Saunders and *Alaria esculenta* (Linnaeus) Greville are now cultivated widely—but not evenly—across their ranges (Peteiro et al. 2016; Stévant et al. 2017; Walls et al. 2017; Bak et al. 2018). The total production—100 s tonnne per annum (authors estimate)—is still meagre compared to the well-established–cultivated kelp species in East Asia (Buschmann et al. 2017).

Cultivation methods that allow the cost-effective production of biomass are essential to improve the economic case for cultivation and encourage greater uptake of macroalgal aquaculture across Europe (van den Burg et al. 2016). In particular, seeding method and deployment have been highlighted as a significant component of the cost of production (Bak et al. 2018). The traditional method of seeding ropes is to rear juveniles attached to twine within a hatchery and then deploy with a dense coverage of 2–10-mm juveniles after 6–8 weeks (Forbord et al. 2012; Kerrison et al. 2016); although, this varies between cultivators. The twine is then wrapped helically around a carrier rope at the seaweed farm. Alternatively, short lengths of twine or individual sporophytes can be selected and inserted into the rope lay (Freitas et al. 2016). A new method, which is currently being developed, uses juvenile sporophytes (0.1–2 mm) which are suspended within a hydrocolloid binder (Kerrison et al. 2018b). This binder is spread onto/embedded into a rope, net or textile sheet immediately prior to submersion in the sea. This method has advantages over twine-seeding by (a) substantially reducing the hatchery space requirements, as there is no need to prepare and maintain twine spools and (b) reduces the deployment time, as there is no need to wrap twine around the rope. However, the reliability and effectiveness of binder-seeding still need to be demonstrated (Kerrison et al. 2018b; Forbord et al. 2019).

A new range of seaweed cultivation materials have been developed by AtSeaNOVA, BE (previously ATSEA Technologies, BE), from trials conducted under the AT~SEA project (2012–2015; FP7 grant no. 280860). Some of these have already been published (Kerrison et al. 2017, 2018a, b, 2019a, b). AlgaeRope is a synthetic polymer rope which comes in either a twisted or braided form produced in 12- and 18-mm diameter. The AlgaeRibbon is a synthetic polymer non-woven of a 50-mm width and 2.3-mm thickness. More information can be found on the AtSeaNOVA website. This study aims to compare the effectiveness of seeding using the established twine- or novel binder-seeding onto either Algaerope or Algaeribbon (AtseaNOVA, BE) in two kelp species. Effectiveness will be assessed by measuring the density and growth morphology of sporophytes established. These characteristics are important for commercial cultivators.

## Materials and methods

The seedstock used for the experiment were clonal gametophytes of *Saccharina latissima* and *Alaria esculenta* maintained long term in F/2-Si medium (Guillard 1975) at 10 °C under red light ca. 20 μmol photons m^−2^ s^−1^, 12:12 (L:D). These gametophytes were locally sourced from the Sound of Kerrera (56.3822° N, − 5.5359° E). Separately in each species, male and female gametophytes from eight to ten parents were homogenised together using a hand blender. Gametophytes between 5 and 65 μm were separated by filtration and resuspended in a fresh F/2-Si medium. The culture was transferred to a white light (same intensity and light cycle) for 3 weeks where oogonia and sporophytes were present in both species. For each species, half of the gametophyte suspension was seeded onto a 100-m spool of polyester twine (2 mm ø; Tecnología Redera Sl, Estonia) and cultured for 8 weeks under optimised hatchery conditions (Kerrison et al. 2016). This allowed the sporophytes to grow up to 15 mm in length. The other half of the gametophyte suspension was maintained in bubbled culture in a 5-L Erlenmeyer flask as previously described (Kerrison et al. 2018b). After 8 weeks, sporophytes between 65 and 1000 μm were separated by filtration then resuspended in a fresh F/2-Si medium containing 1% binder (AtSeaNOVA, Belgium).

Three growth materials were deployed for the experiment on the 20 February 2016 at the Port a’ Bhuiltin seaweed farm (56.4886° N, − 5.4698° E). Firstly, the hatchery seeded twine was wrapped helically around 45 m of 12 mm ø Seasteel rope (Gael Force Marine, UK), held at a 1.5-m depth. Secondly, the sporophyte suspension was binder-seeded at a density of 10,000 m^−1^ onto 45 m of both: (a) 18 mm ø braided AlgaeRope and (b) a 50-mm width AlgaeRibbon (AtSeaNOVA) on the deck of a boat. Within 10 min of seeding, these materials were deployed horizontally into the water at a 1.5-m depth.

Seaweed data was collected over six time-points, with the experiment ending on 15 August 2016. At each time-point, all macroalgae were removed and bagged from five replicate 30-cm sections at randomised locations on the growth materials. Sampling locations were spaced 1 m apart. Each sample (3 treatments × 2 species × 5 replicates) were then spun dry (28,009 W; White Knight Appliances, UK) and weighed to 0.1 g (Student Scales; Philip Harris Ltd., UK) to determine the fresh mass. The samples were immediately frozen for further analysis. Over the following months, bags were defrosted overnight. The total thallus length of all sporophyte > 10 mm was recorded from each bag. The morphology of the five largest sporophytes was also recorded: frond maximum length, frond maximum width, stipe length, % biofouling (visual estimate) and mass of the complete thalli (including biofouling). It should be noted that after defrosting, ~ 20–30% of kelp mass separates into liquor. Therefore, this mass measurement—hereafter referred to as defrosted biomass—underestimates the individual thalli mass. It is included to examine comparative changes and should not be viewed as an accurate representation of the original thalli mass.

Temperature was logged at 30-min intervals at a 1.5-m depth (Onset Computer Corp., USA; UA-002-64). Triplicate 50-mL water samples for nutrient analysis were immediately filtered at 0.4 μm then frozen upon return to the lab. Nutrient analysis was performed using a Lachat 8500 Flow Injection autoanalyser (Hach Lange Ltd., UK) using the manufacture’s methods.

## Statistics

A general linear model (GLM) analysis of variance was used to test for statistical differences between species, conditions and time-points using Minitab 18.1 (LEAD Technologies, USA). All data were square-root-transformed pre-analysis, and the normality and homogeneity of variance of the data were tested (Anderson and Darling 1952; Levene 1960). In some instances, inequality of variance was still present; yet, analysis of variance is quite robust to such deviations when sample sizes are balanced (Sokal and Rohlf 1995).

## Results

### Physicochemical conditions

**Fig. 1:**
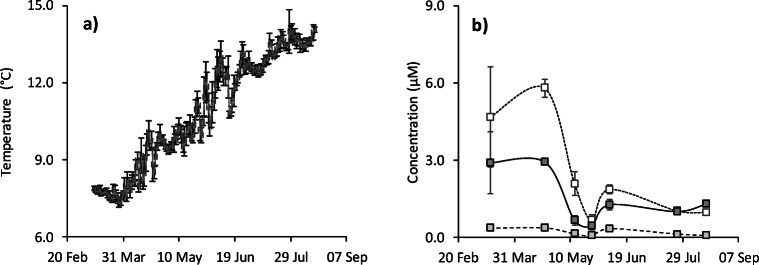
Physicochemical conditions at the Port a’ Bhuiltin seaweed farm, UK during the study period. **a** Temperature shown as daily mean ± standard deviation (*n* = 47). **b** Macronutrient concentrations of nitrate+nitrite (white square, dotted line), silicate (dark grey square, solid line) and phosphate (light grey square, dashed line); All shown as mean ± standard deviation (*n* = 3)

The water temperature rose from a minimum of 7.5 °C at the end of March, to a maximum of 14 °C at the end of the study period (Fig. 1a). The macronutrient nutrient concentrations were stable between March and May: 5 μM nitrate+nitrite, 3 μM silicate and 0.4 μM phosphate (Fig. 1b). Each concentration declined by ca. 85% by 25 May to 0.7, 0.4 and 0.06 μM, respectively. The concentrations then stabilised over the summer at 1.3, 1.2 and 0.2 μM, respectively.

### Biomass yield and sporophyte density

**Fig. 2:**
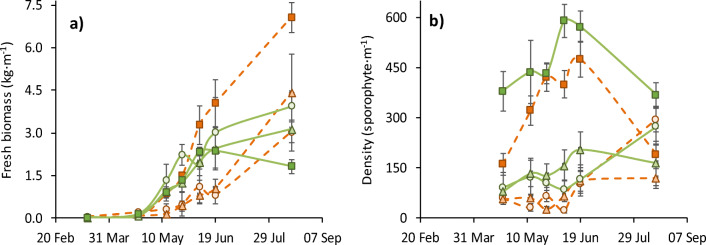
Growth parameters of *Saccharina latissima* (orange dotted line) and *Alaria esculenta* (green solid line) cultivated at the Port a’ Bhuiltin seaweed farm using three methods: twine-seeded longline (light shade circle) binder-seeded Algaerope (medium shade triangle) and binder-seeded Algaeribbon (dark shade square, solid line). **a** Fresh biomass yield. **b** Density of sporophytes > 1 cm. Shown is mean ± standard deviation/2 (*n* = 5)

The biomass yield was significantly different between species (GLM: *F*_**1**,2,5171_ = 5.7, *P* < 0.02). *Alaria esculenta* frond growth plateaued in June, whereas in *S. latissima* it continued to increase into August (Fig. 2a). The growing method did not significantly affect the *A. esculenta* biomass yield (*P* > 0.05); although, when examined alone, the final yield (15 August) was significantly lower on the direct seeded ribbon (AN: *F*_**2**,12_ = 5.1, *P* < 0.05). Growing method did affect the yield of *S. latissima* (GLM: *F*_1,**2**,5,82_ = 42.9, *P* < 0.0001): Ribbon grown *S. latissima* had a far higher yield, 4-fold higher than the other growth materials in June.

### Physical observation of the growth materials

It was noticed that after the first few months, the AlgaeRope had elongated and drooped deeper than 1.5 m. The rope was re-tensioned in 7 June 2016. This is expected to have negatively affected the development of the seaweed before this point. Both the twine-longline and AlgaeRibbon remained at the correct depth and tension. It was noted that > 95% of the developing sporophytes on the AlgaeRibbon grew on only one plane of the rope.

Sporophyte density was higher in *A. esculenta* (GLM: *F*_**1**,2,5171_ = 26.3, *P* < 0.0001) and 4–5-fold higher on binder-seeded ribbon (Fig. 2b). The sporophyte density increased over the study period (GLM: *F*_1,2,**5**171_ = 5.9, *P* < 0.0001) in all conditions except on ribbon where it substantially decreased from 19 June to 15 August.

**Fig. 3:**
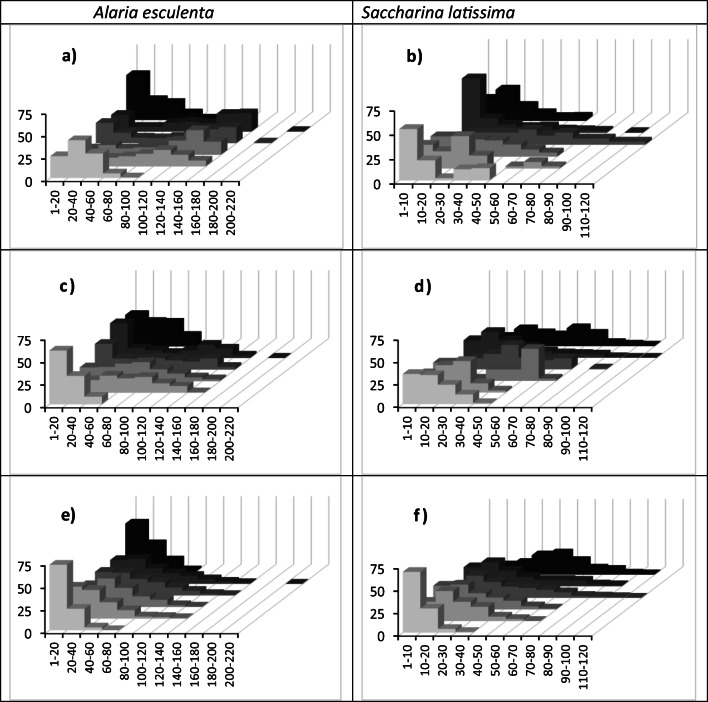
Frequency distribution of total thallus length in *Alaria esculenta* (left column) and *Saccharina latissima* (right column) cultivated at the Port a’ Bhuiltin seaweed farm over six time-points in 2016: (from light to dark grey) 22/04, 13/05, 25/05, 07/06, 19/06 and 15/08. Three methods are shown: twine-seeded longline (**a**, **b**); binder-seeded Algaerope (**c**, **d**) and binder-seeded Algaeribbon (**e**, **f**)

The frequency distribution of thallus lengths was different between the species, which is a reflection of the longer fronds in *A. esculenta*. The frequency distributions appear similar on both the twine-longline and binder-seeded rope (Fig. 3a–d; *P* > 0.05). In these treatments, the median length increased until the end of May. During June, juveniles up to 20 cm became the most common length. The length distribution was different on the binder-seeded ribbons (Fig. 3e–f), where the median remained fairly static at < 40 cm in *A. esculenta* and < 20 cm in *S. latissima*, with the exception of the final *S. latissima* time-point where it rose to 40–50 cm.

### Thallus morphology—species comparison

**Fig. 4:**
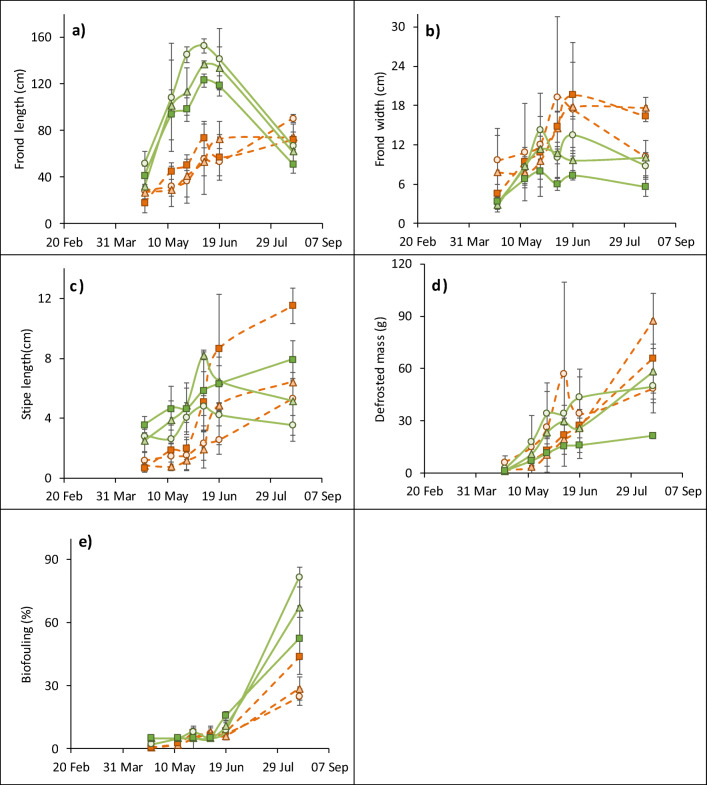
Morphological parameters of *Saccharina latissima* (orange dotted line) and *Alaria esculenta* (green solid line) cultivated at the Port a’ Bhuiltin seaweed farm using three methods: twine-seeded longline (light shade circle) binder-seeded Algaerope (medium shade triangle) and binder-seeded Algaeribbon (dark shade square, solid line). **a** Maximum frond length. **b** Maximum frond width. **c** Stipe length. **d** Biomass of individual thalli, after being defrosted. **e** Frond biofouling. Shown is mean ± standard deviation (*n* = 5)

The morphology of the species differed over the study period: The fronds of *A. esculenta* grew faster (GLM: *F*_**1**,2,5171_ = 153, *P* < 0.0001), and so were 2–3 times longer than *S. latissima* over May–June (Fig. 4a). After this point, the *A. esculenta* fronds eroded in length by more than half by August, while *S. latissima* continued to increase steadily. *Alaria esculenta* had more narrow fronds (GLM: *F*_**1**,2,5171_ = 48.4 *P* < 0.0001), 45% narrower than *S. latissima* over June to August (Fig. 4b). Stipes were longer in *A. esculenta* (GLM: *F*_**1**,2,5171_ = 53.3, *P* < 0.0001); but, growth stalled in this specie over June, whereas it continued in *S. latissima* throughout the study period (Fig. 4c). The defrosted mass of the individual thalli was not significantly different between the species (*P* > 0.05).

Frond biofouling increased exponentially over the study, rising slowly until June, then dramatically into August (Fig. 4e; GLM: *F*_1,2,**5**171_ = 222.7, *P* < 0.0001). *Alaria esculenta* was more prone to biofouling than *S. latissima* at every time-point (GLM: *F*_**1**,2,5171_ = 64.4, *P* < 0.0001), and ended the study with twice the fouling coverage (32 ± 10 vs. 67 ± 16).

### Thallus morphology—growing method comparison

Thallus morphology was also affected by the growing method. The frond length and width of *A. esculenta* were significantly affected by the growing method (GLM: *F*_1,**2**,5,82_ = 70.2 and 23.2, both *P* < 0.0001); longer, wider fronds were found on the twine-longline while those on the binder-seeded ribbon were 23 ± 7% shorter and 42 ± 4% narrower (Fig. 4a, b). The frond morphology of *S. latissima* was not affected by the growing method (*P* > 0.05).

Stipe length was significantly affected in both species: Stipes were longer on the ribbons and shortest on the longline (GLM: *F*_1,**2**,5,82_ = 15.8, *P* < 0.0001). This was especially evident in *S. latissima* ribbon, were the stipes grew > 4-fold (2.0 ± 0.7 to 8.7 ± 3.6 cm) between 25 May and 19 June (Fig. 4c).

The defrosted mass of individual thalli was not significantly affected by the growing method in *S. latissima* (*P* > 0.05), while it was in *A. esculenta* (GLM: *F*_1,**2**,5,82_ = 32.7, *P* < 0.0001), which were 57 ± 5% lighter than from the other growing methods (Fig. 4d).

The growing method did not significantly affect frond biofouling on *A. esculenta* (*P* > 0.05)*,* but *S. latissima* fouling was higher on the ribbon (GLM: *F*_**2**,5,82_ = 3.6, *P* < 0.05), ending the study with 44 ± 8% compared with 25–29% using the other growing methods (Fig. 4e).

## Discussion

The recent development of the direct binder-seeding method could allow greater efficiency in the hatchery and deployment phase of kelp cultivation, reducing biomass production costs (Kerrison et al. 2018b). The three growth materials trialled for kelp cultivation in this study provide a comparison of the typical twine-longline method with the contemporary binder-seeding method onto specially designed textiles (AlgaeRope and AlgaeRibbon). We show that the binder method can yield an equivalent (*A. esculenta*) or higher (*S. latissima*) biomass yield compared to twine-seeded longline. An equivalent yield between twine-longline and binder-seeded textile was previously reported in *S. latissima*, deployed over a similar time-period in the previous year (Kerrison et al. 2018b). Yet, this positive result should be balanced against the current unreliably of the binder method compared to twine-seeding. Previous trials have found the method to be ‘hit or miss’ either very successful or a complete failure (Macleod, A. unpublished results). It is currently hypothesised that this is due to the sporophytes being washed off the substratum, before they can attach, under high wave energy conditions.

The present study is also a demonstration that the binder method can allow almost immediate deployment into the sea, within 10 min post-seeding. This could be useful for the development of future mechanisation, as growth materials can be seeded on boats at the site, rather than being seeded and transported from land. It should be noted that the AlgaeRope growth may have under-performed due to the rope stretching and sinking lower in the water column. This was a particular problem as the rope had a hollow core; a solid core has now been included to limit stretching in the current AlgaeRope product (A. Martinez; AtseaNOVA pers. comms.)

The results of the present study should be considered alongside the recent Norwegian study by Forbord et al. (2019). They found that *S. latissima* twine grown from meiospores in a hatchery for 42 days gave superior performance at sea (longer fronds and higher biomass yield), compared with either gametophyte grown hatchery twine- or sporophyte (binder)-seeded rope, which had the poorest performance in their study. The authors suggest that this is because binder-seeded sporophytes must invest energy into initial holdfast development which slows their early frond growth after deployment. Yet, they also seeded much smaller sporophytes of mean length 45–120 μm (depending on deployment) than in the present study (65–1000 μm). Forbord et al. (2019) recognised that sub-optimal methods used for their sporophyte culture preparation may have slowed the development of the sporophytes prior to deployment. In contrast, their meiospore-seeded twine was exposed to higher light and continuous water refreshment (after 3 days of hatchery culture), so achieving large sporophytes (5–10 mm, see their Fig. 3) prior to deployment. Forbord et al. (2019) demonstrates that hatchery seeded twine may always provide the juveniles with an initial size advantage over binder-seeded material. However, the present study demonstrates that this advantage may become undetectable with improved pre-deployment culturing and/or when farmed under different physicochemical conditions.

### Comparing growing methods

The twine- and binder-seeding methods are fundamentally different with regard to the microenvironment inhabited by the juvenile sporophytes following deployment. In the twine method, a visibly dense culture of attached juvenile sporophytes grow firmly attached to the twine fibres, after spending 6–8 weeks growing in controlled hatchery conditions. This twine is then deployed at the farm by being wrapped helically around a synthetic polymer rope. Their firm initial attachment allows the sporophytes to immediately tolerate high wave energy. The physical and chemical characters of the twine dictate how strongly the juveniles are attached, and so can influence the final yield (Kerrison et al. 2019b). As the sporophytes mature, their developing holdfasts spread onto the carrier rope and will mechanically interlock around the rope and neighbouring holdfasts (pers. obs.). In the present study, we utilised a carrier rope widely used in commercial fishing and other marine applications. It was smooth with a high water contact angle, making it a poor surface for the holdfast bioadhesive attachment (Kerrison et al. 2018a, 2019a). We previously trialled binder-seeding directly onto this rope but this was unsuccessful (unpublished results).

The AlgaeRope and AlgaeRibbon have been designed to provide a large, textured surface with suitable chemistry to enhance the attachment of sporophytes during binder-seeding. The composition is the intellectual property of AtSeaNova BV. Binder-seeded sporophytes are initially unattached and must develop a holdfast attachment to the underlying material, while in situ at the farm. This may slow their early frond development (Forbord et al. 2019). The high viscosity of the binder prevents the seeding sporophytes from being washed away too quickly (Kerrison et al. 2018b). In the present study, the AlgaeRope had a very similar sporophyte density to the twine-longline with only 1–1.5% of the 10,000 sporophytes m^−1^ seeded onto the material retained. This is twice the density achieved on *S. latissima* lines cultured in the Faroe Islands (Bak et al. 2018). The AlgaeRibbon retained only about 5% of the seeded sporophytes, with a similar density to a recent growth trials of *Saccharina latissima forma angustissima* cultured in Maine, USA (Augyte et al. 2017) and *S. latissima* lines grown in Spain and Norway (Peteiro et al. 2006; Peteiro and Freire 2013; Forbord et al. 2019). The ideal seeding density is currently unknown and may vary with end use (discussed further below).

The holdfast of the sporophytes seeded within the Binder must create a firm attachment to the underlying substrate before they are detached due to water motion. The attachment time is currently not published, and we recommend that deployment is planned during a week-long period of calm weather to ensure successful attachment. Many of the seeded sporophytes will be incorrectly oriented, and so the seeding density used in this study was chosen to be far in excess of the maximum amount required. Determining the optimal seeding density and methods to increase the retention of the seeded sporophytes are needed to make the binder-seeding method applicable widely to the European cultivation industry.

### Differences in thallus morphology

The morphology of the seaweed thallus is very important when assessing the quality of the biomass for human food (Kawashima 1993; Peteiro and Freire 2011). Larger individual thalli are generally preferred, as they are easier to inspect and clean. Thicker fronds (higher substantiality value) are also favoured for food use (Peteiro et al. 2006). Differences were seen in thallus morphology between the treatments, which we believe is mainly due to their different sporophyte densities. Density-dependent growth in seaweed has been well described, with slower individual growth at higher density (Edwards and Connell 2012). The very high density of sporophytes on the AlgaeRibbon caused intraspecific space competition between neighbours (Reed et al. 1991) leading to changes to the thallus morphology. Frond morphology is known to respond plastically to the water flow environment, with strap-like blades in high flow and wide ruffled “undulate” fronds in low flow due to longitudinal tension (Gerard et al. 1987; Koehl et al. 2008; Nanba et al. 2011); but as far as the authors are aware, such morphological changes have not been demonstrated previously regarding density.

In the current study, the lines were cultivated under the same background environmental conditions; yet, some difference in the local physicochemical environment of the individual kelp blades must be responsible for the observed morphological change: The high-density sporophytes will have greater competition or light, nutrients and CO_2_ and will also modify the surrounding hydrodynamic environment. Alternatively, they may be responding to a signal of a crowded environment, e.g. physical contact with neighbours, higher pH or an aqueous signalling molecule. It is tempting to hypothesise that the longer stipes seen at higher sporophyte density are an adaptive response to escape the competition for resources by stretching into open space, similar to what is seen in land plants (Gruntman et al. 2017). Intraspecific competition appeared particularly intense in *A. esculenta* where fronds were substantially narrowed by 45% and resource limitation had reduced their individual growth rates.

These morphology changes have implications for the end-use of the biomass. Larger individual fronds, with shorter stipes are generally favoured for fresh food applications; therefore, a low-density seeding method is preferred. Density is carefully controlled in East Asian kelp cultivation intended for human consumption, to reduce intraspecific competition and maximise individual frond size (FAO 1989). Alternatively, for biorefinery applications, maximising the final biomass yield is favoured, and so a high-density seeding may be preferable in *S. latissima* but may not be appropriate in *A. esculenta* where biomass yield was lower. High-density seeding results in a skewed population distribution, with small thalli making up the majority, while the largest individuals have longer stipes and may have smaller fronds. The biochemical implications of these morphology changes on the harvested biomass have not been studied, e.g. if the desired compound is only present within the stipe, a higher content of stipe will be preferred. Further study is needed on the impact of seeding density on the seasonal changes in seaweed growth biochemistry (Schiener et al. 2015).

Similar to thallus morphology, biofouling can also reduce the value of cultivated seaweed, particularly for food (Lüning and Mortensen 2015). The seasonal development of biofouling varies with site and geographical region, but typically—as in the present study—becomes problematic from May–June onward (Buck and Bucholz 2004; Lüning and Mortensen 2015; Førde et al. 2016; Walls et al. 2017; Matsson et al. 2019). This the first report that *A. esculenta* is more susceptible to biofouling that *S. latissima*, and so will require an earlier harvest, particularly for food applications. Interestingly, fouling was greater in the higher seeding density on the ribbon, but only in *S. latissima*. This again appears to suggest that lower density growth should be favoured when cultivating for food.

Holdfast morphology data was not recorded in this study, but some observations were noted. The holdfast of *A. esculenta* develops as a small button (up to ø 20 mm when fully grown) allowing many compatriots to develop side-by-side. In contrast, the holdfast of *S. latissima* forms adventurous hapterae (each can be upwards of 5 cm in length) which spread over the available substratum. We therefore would expect greater holdfast competition, and so self-thinning in *S. latissima*. This may be responsible for the particularly large reduction in sporophyte density seen on *S. latissima* ribbons at the end of the study period.

The *S. latissima* hapteron development appeared different on the AlgaeRibbon or rope surface. On the ribbon, the holdfasts appeared more compact and hardened, while on the rope, a tangled mass of soft overlapping hapterae formed. The reason for this difference will be explored in a further paper (Kerrison, P.D. unpublished results), yet this has implications regarding the current study on seaweed production. Firstly, a large mass of hapterae will require a larger energy investment, potentially reducing frond growth. Secondly, the biochemical composition of soft or hard holdfasts will be different. And finally, a hardened holdfast is more difficult to remove from the surface (pers. obs), for harvesting and reuse of the growth material before reseeding.

### Growth cycle of *A. esculenta* and *S. latissima*

Rapid growth of the seaweed was observed during the spring, which is well described in the Laminariales (Parke 1948; Kain 1979). However, growth in *A. esculenta* appears to stall in June, whereas it continues in *S. latissima*. This is confirmed by biomass yield, defrosted biomass, frond length, frond width and stipe length. This stall in growth, and the higher susceptibility of *A. esculenta* to biofouling, fits with the industry view that *A. esculenta* should be harvested earlier than *S. latissima*, although the exact timing will be site dependent. In both species, distal frond erosion was evident during the summer, but was not quantified. In *A. esculenta*, this led to a reduction in frond length from June onward.

## Conclusions

This study has shown that the binder-seeding method can produce a similar or higher biomass yield during the harvesting period, when compared to the widespread twine-longline method. Yet, due to differences in seeding density, morphological changes were observed, and the length distribution of the populations were very different. These differences are expected to impact on the biochemical composition of the crop. The growth method should be varied depending on the end use of the biomass.
